# A proof-of-concept study on endoscopic ultrasound-guided biopsy of detrusor muscle in porcine bladders

**DOI:** 10.3389/fonc.2023.1160463

**Published:** 2023-06-02

**Authors:** Jeremy Yuen-Chun Teoh, Chak-Lam Cho, Ronald Cheong-Kin Chan, Kang Liu, Hongda Zhao, Gianluca Giannarini, Dmitry Enikeev, Chi-Fai Ng, Anthony Yuen-Bun Teoh

**Affiliations:** ^1^ S.H. Ho Urology Centre, Department of Surgery, The Chinese University of Hong Kong, Hong Kong, Hong Kong SAR, China; ^2^ Urothelial Carcinoma Working Group, European Association of Urology – Young Academic Urologists (EAU-YAU), Arnhem, Netherlands; ^3^ Department of Anatomical and Cellular Pathology, The Chinese University of Hong Kong, Hong Kong, Hong Kong SAR, China; ^4^ Urology Unit, “Santa Maria della Misericordia” University Hospital, Udine, Italy; ^5^ Institute for Urology and Reproductive Health, Sechenov University, Moscow, Russia; ^6^ Department of Urology, Medical University of Vienna, Vienna, Austria; ^7^ Department of Surgery, Prince of Wales Hospital, The Chinese University of Hong Kong, Hong Kong, Hong Kong SAR, China

**Keywords:** detrusor muscle, endoscopic ultrasound-guided biopsy, porcine bladder, transurethral resection of bladder tumor (TURBT), diagnosis

## Abstract

**Introduction:**

Conventionally, we rely on transurethral resection of bladder tumour (TURBT) for local staging of muscle-invasive bladder cancer (MIBC). However, the procedure is limited by its staging inaccuracy which may delay the definitive treatment of MIBC.

**Methods:**

We conducted a proof-of concept study on endoscopic ultrasound (EUS)-guided biopsy of detrusor muscle in porcine bladders. Five porcine bladders were used in this experiment. Upon EUS, four layers of tissue including the mucosa (hypoechoic), submucosa (hyperechoic), detrusor muscle (hypoechoic) and serosa (hyperechoic) could be identified.

**Results:**

A total of 37 EUS-guided biopsies were taken from 15 sites (three sites per bladder), and the mean number of biopsies taken from each site was 2.47±0.64. Among the 37 biopsies, 30 of them (81.1%) obtained detrusor muscle in the biopsy specimen. For the per biopsy site analysis, detrusor muscle was obtained in 73.3% if only one biopsy was taken, and 100% if two or more biopsies were taken from the same biopsy site. Overall, detrusor muscle was successfully obtained from all 15 biopsy sites (100%). No bladder perforation was observed throughout all biopsy processes.

**Conclusion:**

EUS-guided biopsy of the detrusor muscle could be performed during the initial cystoscopy session, thus expediting the histological diagnosis and subsequent treatment of MIBC.

## Introduction

A histological diagnosis of muscle-invasive bladder cancer (MIBC) is often needed before deciding on radical treatment such as radical cystectomy and radiotherapy. However, cystoscopic biopsy could only sample tissue from the superficial layer and can hardly diagnose a MIBC. Therefore, transurethral resection of bladder tumor (TURBT) is often performed even in the case of MIBC for local staging purpose (1). This is however limited by its staging inaccuracy as well as possible delay in offering a definitive treatment for MIBC (2–4), not to mention the risks of anesthesia and surgery just for staging purpose (5, 6).

In this study, we investigated the technical feasibility of endoscopic ultrasound (EUS)- guided detrusor muscle biopsy of the porcine bladder. EUS-guided intervention has long been used in many general surgical procedures (7–9). For example, EUS-guided drainage has become a standard treatment for patients with pancreatic pseudocyst (10, 11). In the case of bladder cancer, by using endoscopic ultrasound, the different layers (mucosa, submucosa, detrusor muscle and serosa) of the bladder wall can be identified and detrusor muscle biopsy may be performed safely without the risk of bladder perforation. Local staging is possible without the need of a formal TURBT. By obtaining a histological diagnosis of MIBC upon cystoscopy and EUS-guided biopsy, the decision on radical treatment can be expedited without the worry of over-treatment. We believe this proof-of-concept study carries important implications on the diagnostic and staging pathway of bladder cancer.

## Methods

**Figure 1:**
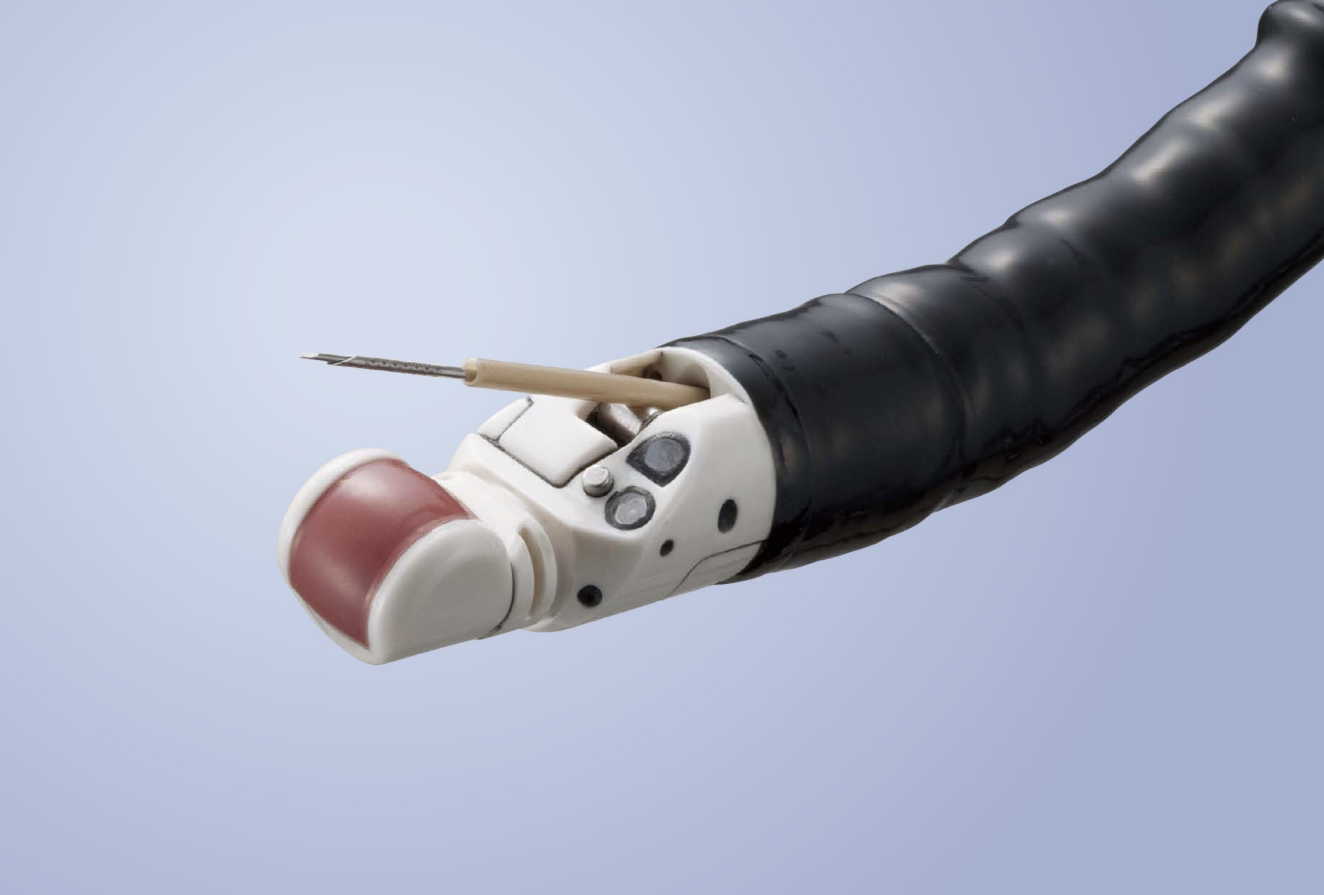
Linear Scanning Ultrasound Endoscope (GF-UCT180).

This is an ex-vivo study investigating the technical feasibility of EUS-guided detrusor muscle biopsy in a porcine bladder model (12). A Linear Scanning Ultrasound Endoscope (GF-UCT180) was used for this experiment (**Figure 1**) (13). The endoscope has an outer diameter of 14.6mm, and a working channel with an inner diameter of 3.7mm. The endoscope is inserted into the bladder via the urethra with saline flow, and the whole bladder can be inspected as in usual cystoscopy. The tip of the endoscope can angulate at four directions; 130 degrees up, 90 degrees down, 90 degrees right and 90 degrees left. The endoscope has a built-in linear ultrasound facing downwards, and an elevator that can control the angle of needle insertion along the plane of the linear ultrasound.

**Figure 2:**
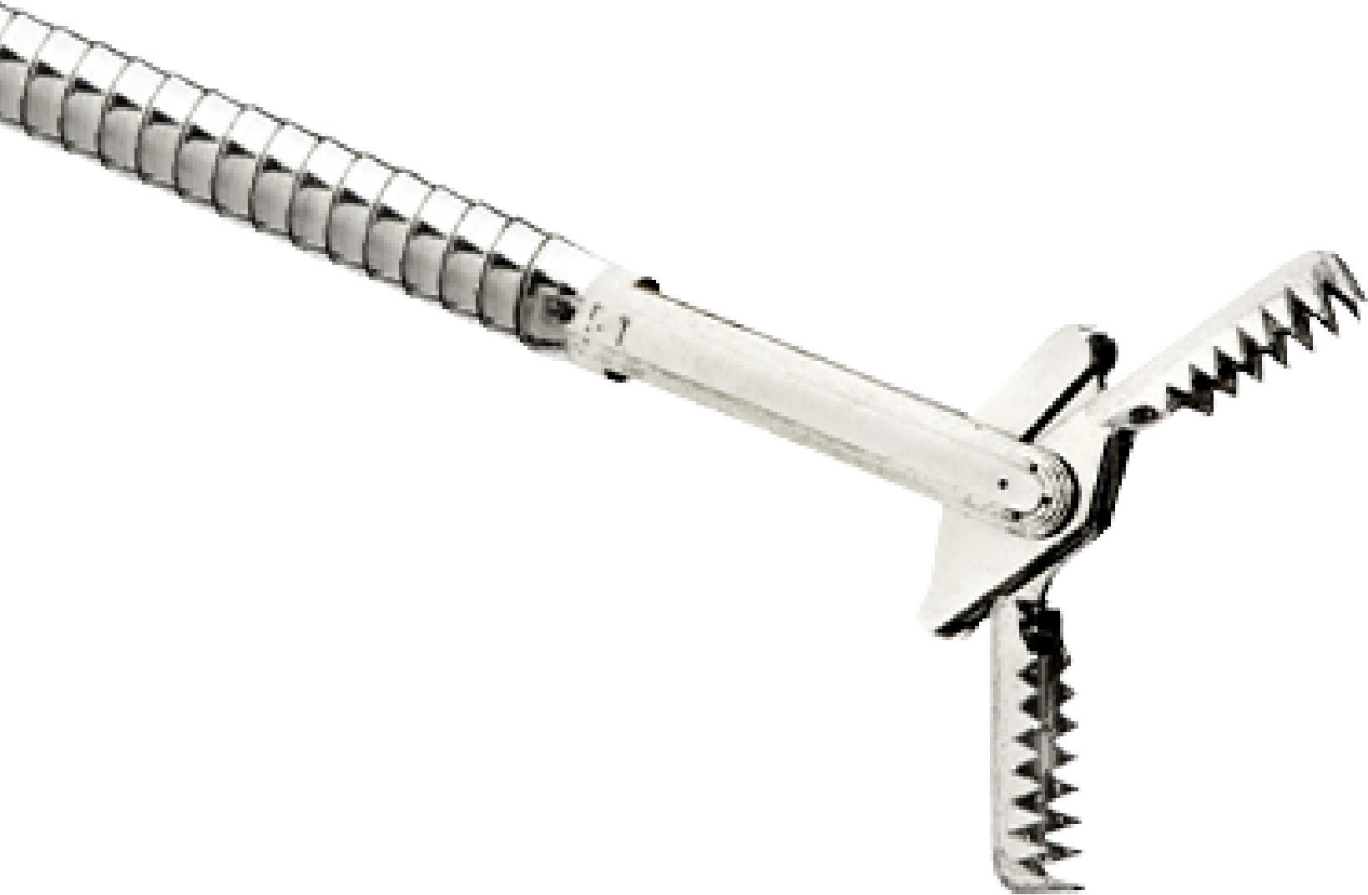
Moray ® Micro Forceps.

In this experiment, EUS was first performed to identify the different layers of the bladder wall. A 19G EZ Shot 3 Plus needle (NA-U220H-8019S) was inserted through the working channel, and into the junction between the submucosa and detrusor muscle under EUS guidance. 3-5ml of normal saline was injected to create a space at the junction. A Moray ^®^ Micro Forceps (0.8mm in diameter, with a jaw opening width of 4.3mm) (**Figure 2**) (14) was then inserted through the lumen of the 19G needle (15), and biopsy was taken from the detrusor muscle layer. A total of five porcine bladders were used. For each porcine bladder, three sites were chosen for biopsy, and for each site, a maximum of three biopsies were taken.

Primary outcome was the presence of detrusor muscle in the biopsy specimen. Per biopsy and per biopsy site analyses were performed. Secondary outcome included the procedural time for each biopsy site.

## Results

**Figure 3:**
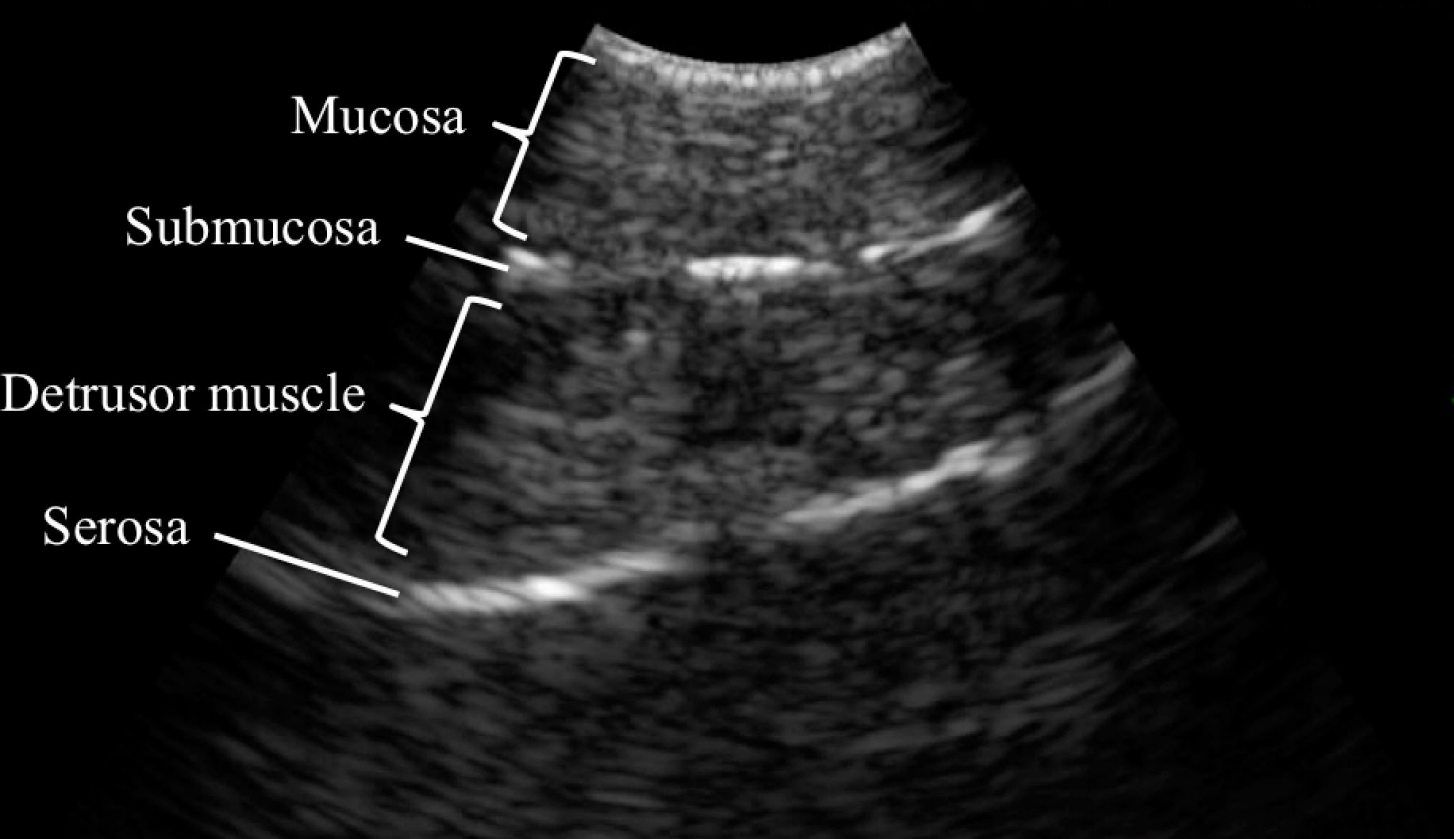
Four layers of tissue.

**Figure 4:**
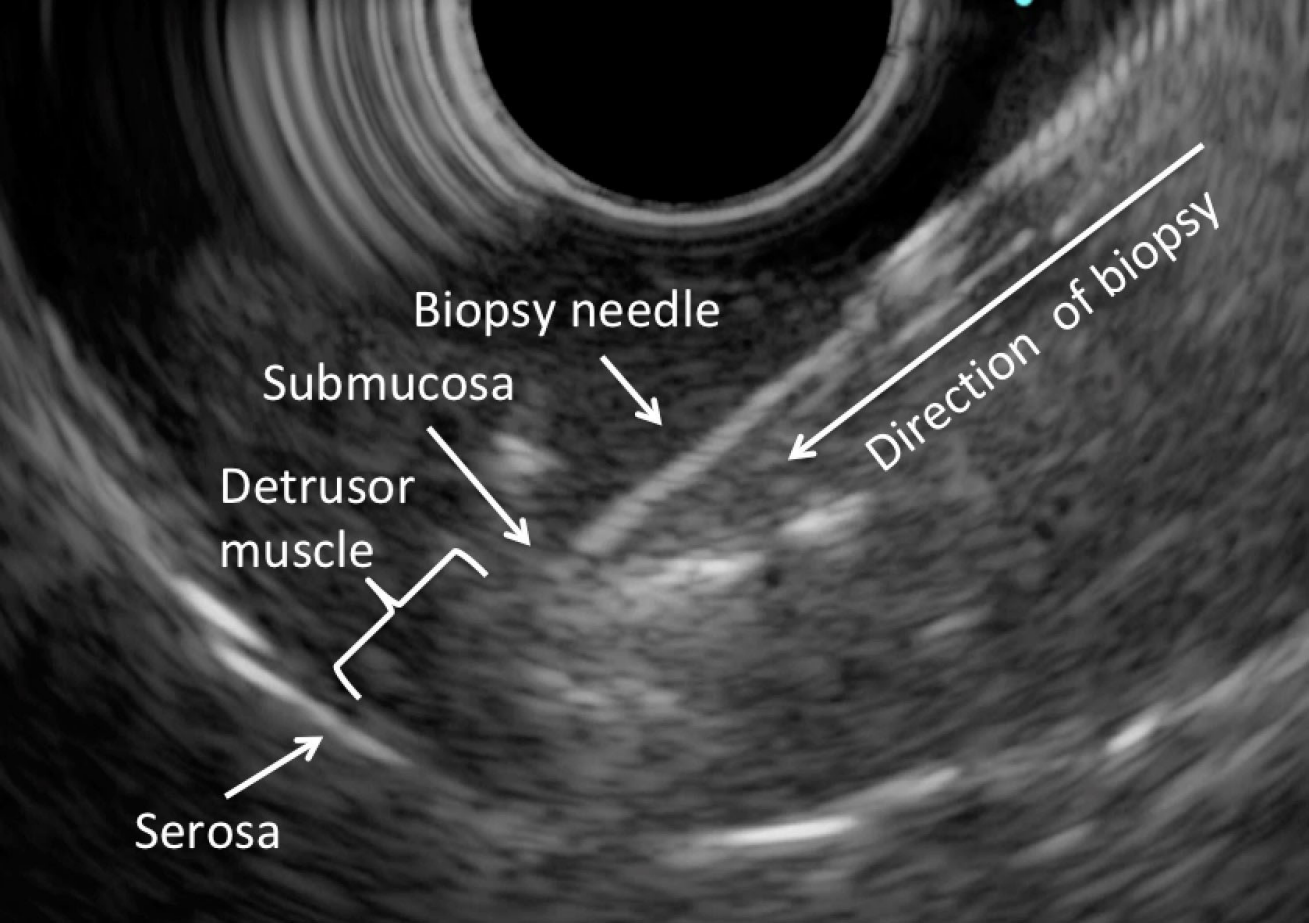
EUS-guided biopsy.

**Figure 5:**
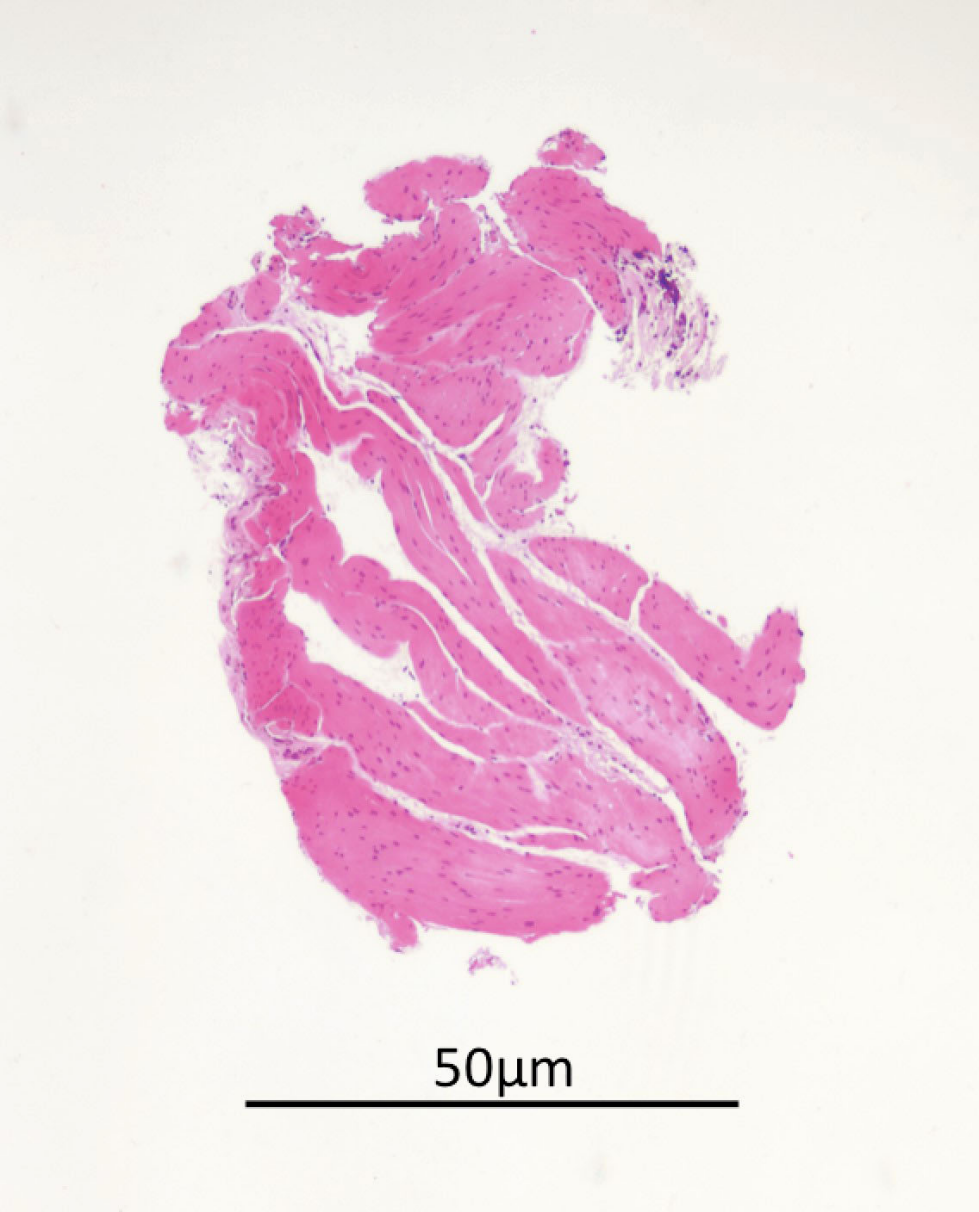
The biopsy specimen.

The whole porcine bladder could be inspected using the Linear Scanning Ultrasound Endoscope. Upon EUS, four layers of tissue including mucosa (hypoechoic), submucosa (hyperechoic), detrusor muscle (hypoechoic) and serosa (hyperechoic) could be identified (**Figure 3**). EUS-guided needle puncture into the junction between the submucosa and detrusor muscle could be performed (**Figure 4**). After normal saline was injected, biopsy of the detrusor muscle could be performed using the through-the-needle forceps. The biopsy specimen could be examined for detrusor muscle under microscopy (**Figure 5**).

A total of five porcine bladders were used in this experiment, and 15 sites for chosen for biopsy (three sites per bladder). The mean procedural time was 5.33 ± 1.21 minutes for each site, and the longest procedural time was 7.01 minutes. A total of 37 biopsies were taken from 15 sites, and the mean number of biopsies taken from each site was 2.47 ± 0.64. Among the 37 biopsies, 30 of them (81.1%) obtained detrusor muscle in the biopsy specimen. For the per biopsy site analysis, detrusor muscle was obtained in 73.3% if only one biopsy was taken, and 100% if two or more biopsies were taken from the same biopsy site. Overall, detrusor muscle was successfully obtained from all 15 biopsy sites (100%) (**Table 1**). No bladder perforation was observed throughout all biopsy processes.

**Table 1:** Per biopsy and per biopsy site analyses on the presence of detrusor muscle.

	Presence of detrusor muscle			
	First biopsy	Second biopsy	Third biopsy	Overall
Bladder A				
Site 1	+	+	NA	+
Site 2	+	+	+	+
Site 3	–	+	+	+
Bladder B				
Site 1	+	+	NA	+
Site 2	+	NA	NA	+
Site 3	–	+	+	+
Bladder C				
Site 1	+	+	–	+
Site 2	+	+	+	+
Site 3	+	+	+	+
Bladder D				
Site 1	–	+	NA	+
Site 2	+	+	+	+
Site 3	+	–	–	+
Bladder E				
Site 1	+	+	NA	+
Site 2	–	+	NA	+
Site 3	+	+	NA	+
**Per biopsy analysis**	73.3% (11/15)	92.9% (13/14)	75% (6/8)	81.1% (30/37)
Per biopsy site analysis				
Based on first biopsy alone	73.3% (11/15)			
Based on first and second biopsies		100% (15/15)		
Based on all three biopsies			100% (15/15)	

NA, Not applicable, biopsy was not taken.

## Discussion

TURBT as a staging procedure has long been criticized for its staging inaccuracy, risks of surgery and potential delay of definitive treatment in the case of MIBC. There is growing interest in diagnosing MIBC based on non-histological parameters, with the aim of proceeding to definitive treatment without the need of a staging TURBT. The Vesical Imaging Reporting and Data System (VI-RADS) score has been proposed to predict the probability of MIBC based on multiparametric MRI, and it has been demonstrated to have a high sensitivity of 0.83 and specificity of 0.90 (16). The BladderPath study aims to compare between the imaging-guided pathway (based on both cystoscopic and MRI findings) and the standard pathway for staging MRI; it is currently underway and the results are eagerly awaited (17). However, the decision to undergo radical cystectomy without histological diagnosis of MIBC is not without risks. The main concern is the possibility of an over-treatment for patients with a final pathology of non-muscle-invasive bladder cancer. In a prospective study of 231 patients undergoing TURBT (18), MRI using a VI-RADS cutoff of ≥3 showed a positive predictive value of 77.5%. The staging performance was better using a higher VIRADS cut-off of ≥4 (18), but still the risk of over-staging could not be eliminated.

To many urologists, having a histological diagnosis of MIBC is still considered a pre-requisite to decide for a major surgery like radical cystectomy (19). Therefore, we take a different approach and investigate an exploratory method which can potentially diagnose MIBC histologically during the initial cystoscopy session. With the use of EUS, biopsy of the detrusor muscle could be performed reliably without the worry of puncturing the serosa. In our study, among the 37 biopsies performed, 30 of them (81.1%) could obtain detrusor muscle in the specimen. With a detrusor sampling rate of 81.1% in a single biopsy, the probability of obtaining detrusor muscle from the same biopsy site would increase to 96.4% if two biopsies were taken, and 99.3% if three biopsies were taken. The longest procedural time was only 7.01 minutes and no bladder perforation was observed. When applied clinically, a minimum of three biopsies from the same biopsy site should be considered to maximize staging accuracy.

While we believe the concept of EUS-guided biopsy of detrusor muscle is sound and compelling, there are technical obstacles that we need to overcome before it can be applied clinically. The EUS endoscope has an outer diameter of 14.6mm and it is too big for human urethra. A similar endobronchial ultrasound (EBUS) bronchoscope (BF-UC180F) (20) has an outer diameter of 6.9mm and is more permissible, unfortunately, the working channel does not allow insertion of the 19G EZ Shot 3 Plus needle (NA-U220H-8019S). The ViziShot FLEX 19G EBUS-TBNA needle (21) can be inserted through the EBUS bronchoscope, but its lumen does not allow introduction of the Moray ^®^ Micro Forceps. In addition, the linear ultrasound of the EBUS bronchoscope is facing upwards (instead of facing downwards in the EUS endoscope) and it is more difficult to orientate during the procedure.

A tailor made EUS cystoscope is urgently needed, until then can we realize this concept of EUS-guided biopsy in patients with bladder tumors. Further human clinical studies will be needed to determine the safety, efficacy and diagnostic performance of EUS-guided biopsy. Should this concept be successful, we envision the future diagnostic pathway of MIBC as follows: 1) Flexible cystoscopy is performed as per usual indications such as painless hematuria, 2) Same session EUS-guided biopsy of the detrusor muscle if a large bladder tumor is detected upon flexible cystoscopy, 3) A histological diagnosis of MIBC can be made without TURBT, and the decision for a definitive treatment can be expedited with any concerns of over-treatment. We believe this is a promising concept that can potentially pave the diagnostic pathway of MIBC in the future.

On the other hand, we must acknowledge the potential limitations of such diagnostic approach. First, although EUS-guided biopsy may optimize staging accuracy of MIBC, we should not neglect the potential benefit of maximal transurethral resection in optimizing subsequent treatment outcomes. Krik et al. reported that maximal transurethral resection may improve pathological response at cystectomy. In this multicenter cohort study, 579 patients who underwent maximal resection had less advanced nodal stage, clinical tumor, and lower rates of positive surgical margins than those with incomplete transurethral resection (22). In MIBC patients who are contemplating chemoradiation, maximal transurethral resection should also be considered as part of trimodality therapy. In a cohort study of 475 patients undergoing trimodality therapy (23), it was found that patients who had complete transurethral resection had better disease-specific survival and overall survival than those who had incomplete transurethral resection. Therefore, following the diagnosis of MIBC upon EUS-guided biopsy, whether transurethral resection is still needed should be discussed and decided in an individualized manner. Second, there is a potential limitation in the detection of variant histology. While EUS-guided biopsy may help diagnose MIBC, the specimen obtained may not be sufficient to reflect the true histology of bladder cancer. The detection of variant histology carries several clinical implications. For example, in the presence of variant histology, neoadjuvant chemotherapy is often considered before radical cystectomy (24). Also, more aggressive treatment in the form of radical cystectomy is usually preferred over radiotherapy (25). The failure of detection of variant histology may therefore compromise the oncological outcomes, and it is a potential risk that we need to consider. Third, the bladder wall anatomy and ultrasound images could differ across different sectors of the bladder. There is a lack of data on this aspect and further studies in human bladders will be needed to define this further. Whether ultrasound alone can differentiate between non-muscle-invasive bladder cancer and MIBC also remains to be explored in future studies.

## Data availability statement

The original contributions presented in the study are included in the article/supplementary material. Further inquiries can be directed to the corresponding authors.

## Ethics statement

The animal study was reviewed and approved by Prince of Wales Hospital, The Chinese University of Hong Kong, Hong Kong, China.

## Author contributions

Conception and design: JT, C-LC. Acquisition of data: JT, KL, HZ. Analysis and interpretation of data: JT, C-LC, RC. Drafting of the manuscript: JT. Critical revision of the manuscript for important intellectual content: GG, DE, C-FN, AT. Statistical analysis: JT. Obtaining funding: JT. All authors contributed to the article and approved the submitted version.
